# Differences in the photosynthetic plasticity of ferns and *Ginkgo* grown in experimentally controlled low [O_2_]:[CO_2_] atmospheres may explain their contrasting ecological fate across the Triassic–Jurassic mass extinction boundary

**DOI:** 10.1093/aob/mcx018

**Published:** 2017-03-11

**Authors:** C. Yiotis, C. Evans-Fitz.Gerald, J. C. McElwain

**Affiliations:** 1 Earth Institute, O’Brien Centre for Science, University College Dublin, Belfield, Ireland; 2 School of Biology and Environmental Science, University College Dublin, Belfield, Ireland

**Keywords:** Triassic–Jurassic boundary, *Ginkgo biloba*, gymnosperms, monilophytes, angiosperms, high CO_2_, low O_2_, photosynthetic plasticity, photorespiration, photodamage, stomatal conductance, mesophyll conductance

## Abstract

**Background and Aims** Fluctuations in [CO_2_] have been widely studied as a potential driver of plant evolution; however, the role of a fluctuating [O_2_]:[CO_2_] ratio is often overlooked. The present study aimed to investigate the inherent physiological plasticity of early diverging, extant species following acclimation to an atmosphere similar to that across the Triassic–Jurassic mass extinction interval (TJB, approx. 200 Mya), a time of major ecological change.

**Methods** Mature plants from two angiosperm (*Drimys winteri* and *Chloranthus oldhamii*), two monilophyte (*Osmunda claytoniana* and *Cyathea australis*) and one gymnosperm (*Ginkgo biloba*) species were grown for 2 months in replicated walk-in Conviron BDW40 chambers running at TJB treatment conditions of 16 % [O_2_]–1900 ppm [CO_2_] and ambient conditions of 21 % [O_2_]–400 ppm [CO_2_], and their physiological plasticity was assessed using gas exchange and chlorophyll fluorescence methods.

**Key Results** TJB acclimation caused significant reductions in the maximum rate of carboxylation (*V*_Cmax_) and the maximum electron flow supporting ribulose-1,5-bisphosphate regeneration (*J*_max_) in all species, yet this downregulation had little effect on their light-saturated photosynthetic rate (*A*_sat_). *Ginkgo* was found to photorespire heavily under ambient conditions, while growth in low [O_2_]:[CO_2_] resulted in increased heat dissipation per reaction centre (*DI*_o_/*RC*), severe photodamage, as revealed by the species’ decreased maximum efficiency of primary photochemistry (*F*_v_/*F*_m_) and decreased *in situ* photosynthetic electron flow (*J_situ_*).

**Conclusions** It is argued that the observed photodamage reflects the inability of *Ginkgo* to divert excess photosynthetic electron flow to sinks other than the downregulated C_3_ and the diminished C_2_ cycles under low [O_2_]:[CO_2_]. This finding, coupled with the remarkable physiological plasticity of the ferns, provides insights into the underlying mechanism of Ginkgoales’ near extinction and ferns’ proliferation as atmospheric [CO_2_] increased to maximum levels across the TJB.

## INTRODUCTION

The end of the Triassic marked the beginning of a period of geological and ecological upheaval known as the Triassic–Jurassic mass extinction event (approx. 200 Mya). Although several studies have questioned the high rates (Hallam, 2002; Tanner *et al.*, 2004) and sources (Bambach *et al.*, 2004) of biodiversity loss across the Triassic–Jurassic boundary (TJB), it is widely considered as the third greatest mass extinction in the Phanerozoic (last approx. 450 million years) (Benton, 1995; McElwain and Punyasena, 2007). Plant communities were severely affected in terms of species turnover rates (Harris, 1937; Visscher and Brugman, 1981; Fowel *et al.*, 1994; McElwain *et al.*, 1999, 2007, 2009; Olsen *et al.*, 2002) and evenness (i.e. the equality of relative abundances among taxa, McElwain *et al.*, 2007, 2009), yet the available data suggest that higher taxonomic ranks displayed remarkable resilience (Ash, 1986; McElwain *et al.*, 2007; Willis and McElwain, 2013). In general there is a negligible impact of mass extinctions on plants at the family level (Ash, 1986; McElwain *et al.*, 2007; McElwain and Punyasena, 2007). Instead, plant communities undergo structural reformation, which includes substantial changes of families’ relative abundances and distributions and/or in some cases the total loss of growth habits (McElwain *et al.*, 2007, 2009; McElwain and Punyasena, 2007; Bonis and Kuerschner, 2012). In this context, differences in the physiological plasticity between species, families or reproductive groups are expected to play a role in shaping the composition of plant communities under changing environmental conditions.

The causal mechanism of the TJB mass extinction has been actively debated (Hallam, 1997; McElwain *et al.*, 1999, 2009; Palfy *et al.*, 2001; Beerling and Berner, 2002; Hesselbo *et al.*, 2002; Olsen *et al.*, 2002; Marzoli *et al.*, 2004). The organic carbon isotope record revealed a major, and synchronous with the mass extinction event, perturbation of the global carbon cycle in the form of a light carbon excursion across the TJB (McRoberts *et al.*, 1997; McElwain *et al.*, 1999; Palfy *et al.*, 2001; Ward *et al.*, 2001; Hesselbo *et al.*, 2002; Guex *et al.*, 2004; Galli *et al.*, 2005; Kuerschner *et al.*, 2007; Williford *et al.*, 2007; Ruhl *et al.*, 2009; Bachan *et al.*, 2012). In addition, recent data strongly support synchroneity between the disruption of the carbon cycle and the early phases of the Central Atlantic magmatic province (CAMP) eruption, pointing towards a cause–effect relationship between extreme volcanism and biodiversity loss (Hesselbo *et al.*, 2002; Marzoli *et al.*, 2004, 2008; Blackburn *et al.*, 2013; Dal Corso *et al.*, 2014). Environmental change associated with CAMP volcanism and possible clathrate release has been tracked across the TJB using fossil plant and soil-based proxy approaches. Stomatal density and stomatal index changes in fossil leaves, for example, have allowed atmospheric CO_2_ to be tracked at high resolution across the TJB, revealing a 2·5- to 4-fold increase at the end of the Triassic (McElwain *et al.*, 1999; Bonis *et al.*, 2010; Steinthorsdottir *et al.*, 2011), which is further corroborated by soil isotope-based estimates (Schaller *et al.*, 2011). Furthermore, tracking the carbon and sulphur cycles, which are controls of atmospheric O_2_, using the isotopic composition of carbonates and sulphur has led to the development of mass-balance models that allow the estimation of O_2_ levels in the atmosphere and reveal that the TJB was also marked by low atmospheric O_2_ (Berner, 2001, 2006).

McElwain *et al.* (1999) suggested a mechanism by which elevated CO_2_ could have functioned as a driver of ecological change by causing a global greenhouse effect with an estimated 4 °C increase of mean annual temperature. They argued that this temperature increase combined with an impairment of the leaf cooling capacity of plants due to high CO_2_-induced reductions in the stomatal aperture and/or stomatal density of the leaves resulted in leaf size-dependent thermal damage (Kramer and Boyer, 1995). Consequently, broadleaved Ginkgoales were replaced by related species possessing more dissected leaves, while at the same time monilophytes (ferns), which possess dissected leaves, proliferated across much of the Northern hemisphere including East Greenland (McElwain *et al.*, 2007), Germany, Sweden (van de Schootbrugge *et al.*, 2009) and North America (Fowell and Olsen, 1993; Olsen *et al.*, 2002).

Despite strong evidence that Earth’s atmosphere and biota have co-evolved throughout Earth history (Woodward, 1987; Tolbert *et al.*, 1995; Beerling *et al.*, 2001; Ehleringer *et al.*, 2005; Igamberdiev and Lea, 2006; Berner *et al.*, 2007; Franks and Beerling, 2009; Willis and McElwain, 2013), our understanding of the mechanisms by which atmospheric composition can shape patterns in plant evolution, such as those documented across the TJB, remains limited. Although several studies have shown that high CO_2_ and sub-ambient O_2_ have differential effects upon different species (Fukao and Bailey-Serres, 2004; Wang *et al.*, 2012; Haworth *et al.*, 2013), few studies have attempted to explain past, major shifts in ecological dominance through the prism of these differences in the physiological plasticity under fluctuating atmospheric O_2_ and CO_2_.

Due to the dual oxygenation/carboxylation activity of plants’ primary carboxylase (i.e. Rubisco), every change in the relative abundance of [O_2_] and [CO_2_] in the atmospheric mixture results in corresponding changes in the enzyme’s carboxylation and oxygenation rates, which are accompanied by dynamic adjustments of the energy flows that support them with ATP and reducing power in the form of NADPH (Farquhar *et al.*, 1980; von Caemmerer, 2000). Since all the light energy absorbed by a plant needs to be quenched, a fine balance between the energy and the reducing power produced during the ‘light reactions’ and those consumed at the ‘dark reactions’ of photosynthesis and photorespiration is essential (Sharkey, 1990; Zhang and Portis, 1999; Andersson, 2008; Parry *et al.*, 2008). However, acclimation to high CO_2_ is known to result in a wide range of morphological and physiological adaptations such as the downregulation of Rubisco activity and an increase in the resistances to CO_2_ diffusion (Ainsworth and Long, 2005; Kirkham, 2011; Kitao *et al.*, 2015) that further complicate the necessary fine adjustment of the energy flows within the photosynthetic machinery. Accordingly, the aim of the present study was to address this gap in our knowledge by investigating the responses of plants belonging to all three major plant reproductive grades (angiosperms, gymnosperms and ferns) after exposure to O_2_ and CO_2_ atmospheric concentrations similar to those that prevailed across the TJB. Our hypothesis was that the enhanced resilience of ferns across the TJB low [O_2_]:[CO_2_] bottleneck was, at least partly, a result of their inherent increased physiological plasticity under low [O_2_]:[CO_2_] conditions compared with *Ginkgo*. In particular, our study focused on the ability of the different plant groups to readjust the energy flows within their photosynthetic apparatus effectively so that they match the acclimated rates of Rubisco carboxylation and oxygenation. Our approach did not exclude the angiosperms, even though the general consensus is that they first evolved much later than the TJB, during the Cretaceous (Crane *et al.*, 1995; Soltis and Soltis, 2004). A combined consideration of the generic diversity in angiosperms (Niklas *et al.*, 1983) and the atmospheric levels of O_2_ and CO_2_ (Berner, 2006) reveals that their explosive radiation and final dominance was seemingly unaffected by the rapid increase in the initially low, and similar to that of the TJB, [O_2_]:[CO_2_] ratio. Based on their evolutionary history and ecological success through a wide range of [O_2_]:[CO_2_] ratios, we anticipated that the angiosperms would also display an increased plasticity compared with *Ginkgo* under simulated low [O_2_]:[CO_2_] atmospheric conditions.

## MATERIALS AND METHODS

### Plant material

Mature plants from five species belonging to some of the most early diverging families within each major reproductive grade (Supplementary Data Table S1), namely the monilophytes *Osmunda claytoniana* L. and *Cyathea australis* (R. Brown) Domin, 1929, the gymnosperm, ‘living fossil’ *Gingko biloba* L., and the angiosperm species *Drimys winteri* J.R. Forst. & G. Forst and *Chloranthus oldhamii* Solms were purchased, repotted and then acclimated to glasshouse conditions (mean temperature = 18 °C) at University College Dublin Rosemount Environmental Research Station glasshouses for approx. 2 weeks. Pot size, soil mixture, type and amount of fertilizer used were determined independently based on the size, age and special preferences of each species (Table S1).

### Controlled-environment experiments

A trial study to assess potential ‘chamber effects’ was conducted prior to the initiation of growth chamber experiments (Porter *et al.*, 2015). The study revealed that some of the eight Conviron BDW40 (Winnipeg, Manitoba, Canada) walk-in growth chambers of University College Dublin’s PÉAC facility (Rosemount, University College Dublin) display significant ‘chamber effects’, thus their further use in the present study was avoided. Following the 2 week acclimation under glasshouse conditions, 2–3 fully expanded leaves were tagged on each individual and the plants were then transferred into four BDW40 growth chambers that displayed no chamber effects (see Porter *et al.*, 2015). Half of the plants were subjected to a replicated TJB atmospheric treatment in two of the chambers for 2 months, while the rest were grown under ambient atmospheric conditions for the same amount of time, serving as our controls (Supplementary Data Table S2). The atmospheric composition used for the TJB treatment was 16 % O_2_ and 1900 ppm CO_2_, which is a good approximation of the corresponding mean values for atmospheric O_2_ and CO_2_ across the boundary reported by mass balance modelling and palaeo-proxy studies (McElwain *et al.*, 1999; Berner, 2001, 2006; Bergman *et al.*, 2004; Belcher and McElwain, 2008; Bonis *et al.*, 2010; Steinthorsdottir *et al.*, 2011). The O_2_ and CO_2_ concentrations used for the ambient treatment were 21 % and 400 ppm, respectively (Table S1).

CO_2_ in each chamber was monitored by a WMA-4 infrared analyser (PP-Systems, Amesbury, MA, USA), and injection of compressed CO_2_ (BOC Gases Ireland Ltd, Bluebell, County Dublin, Ireland) enabled stable within-chamber CO_2_ concentrations well above ambient levels. The O_2_ concentration in each chamber was monitored by a PP-systems OP-1 oxygen sensor, and injection of compressed N_2_ produced by a nitrogen generator (Dalco Engineering, Dunshaughlin, County Meath, Ireland) was used to reduce the O_2_ levels below ambient. The rest of the growth conditions were kept constant between the two treatments. All plants were grown under a 16 h/8 h simulated day/night program; 05·00–06·00 h, dawn; 06·00–09·00 h, light intensity progressively rises from 300 to 600 μmol m^–2^ s^–1^; 09·00–17·00 h, mid-day light intensity of 600 μmol m^–2^ s^–1^; 17·00–20·00 h, light intensity decreases from 600 to 300 μmol m^–2^ s^–1^; 20·00–21·00 h, dusk. Temperature ranged from a night-time low of 15 °C to a mid-day high of 20 °C, and relative humidity was kept constant throughout the day at 65 %. Chamber conditions in all chambers were recorded at 5 min intervals and are summarized in Table S2. Plants were watered regularly, receiving amounts of water which depended on the particular needs of each species under the two separate growth regimes. Upon completion of the 2 month treatment period, chlorophyll fluorescence and gas exchange measurements were performed on one of the tagged leaves of each plant. Measuring leaves that were fully expanded before the initiation of the treatments meant that we were able to exclude the effects of the simulated TJB atmosphere on the morphology of developing leaves (e.g. adaptation of stomatal density, stomatal index, leaf expansion, etc.). Consequently, our experimental approach enabled us to focus on differences in the innate plasticity/adaptability of the photosynthetic physiology, and specifically the ability to readjust the energy flows in the photosynthetic apparatus efficiently, among our test species after exposure to a low [O_2_]:[CO_2_] air mixture.

### Gas exchange measurements

Upon completion of the 2 month acclimation period, the responses of net assimilation rate to incident light (light curves) and intercellular CO_2_ partial pressure (*A*–*C*_i_ curves) were recorded within the chambers with a CIRAS-2 gas analyser (PP-Systems) attached to a PLC6(U) cuvette fitted with a 4·5 cm^2^ measurement window and a red/white light LED unit. Theoretically, measuring within the TJB chambers at 16 % O_2_ could introduce error in our measurements due to band-broadening effects; however, it has been shown that these effects are negligible even when using O_2_-free air (Loriaux and Welles, 2004). Measurements were performed on intact leaves between 09·00 and 12·00 h to avoid potential mid-day stomatal closure. Air flow, leaf temperature and vapour pressure deficit during both the light and *A*–*C*_i_ curves were maintained at 300 cm^3^ min^–1^, 20 °C and 1·0 ± 0·2 kPa, respectively.

For the light response curves, tagged leaves from 3–4 plants per species and treatment were enclosed in the cuvette and illuminated at either 1200 (*O. claytoniana*, *C. australis* and *C. oldamii*) or 1600 (*G. biloba* and *D. winteri*) μmol m^–2^ s^–1^ until full photosynthetic induction, as judged from three consecutive stable readings of CO_2_ assimilation (*A*) and stomatal conductance (*g*_s_), usually within 30 min. The CO_2_ and O_2_ concentrations used were identical to the corresponding growth values for each treatment (ambient, 21 % O_2_–400 ppm CO_2_; TJB, 16 % O_2_–1900 ppm CO_2_). Light levels were then adjusted from 1200/1600 to 20 μmol m^–2^ s^–2^ in nine/ten descending steps, each witha 3 min duration, which was always adequate to obtain stable *A* readings. The light-saturated photosynthetic rate (*A*_sat_) and saturating light intensity were then calculated according to Norman *et al.* (1992).

For the *A*–*C*_i_ curves, the tagged leaves previously used to acquire photosynthetic light response curves were again enclosed in the cuvette and allowed to equilibrate at 400 ppm CO_2_, growth O_2_ concentration (ambient, 21 % O_2_; TJB, 16 % O_2_) and saturating light intensity (calculated from the light response curves) for 30 min. CO_2_ concentration in the cuvette (*C*_a_) was then stepwise decreased from 400 to 50 μmol mol^–1^ (400, 300, 200, 150, 100 and 50) and then increased from 50 to 2000 μmol mol^–1^ (50, 400, 500, 600, 800, 1000, 1200, 1600 and 2000). Relative stability of *C*_a_ and *A* values at each step typically took 4 min, while a close agreement between the two measurements taken at 400 μmol mol^–1^ indicated that exposure to low *C*_a_ had not affected the activation state of Rubisco (von Caemmerer and Edmondson, 1986; Ethier and Livingston, 2004). The resultant response curves were fitted using the model equations of Long and Bernacchi (2003). Implementation of the model allowed the calculation of the maximum Rubisco-limited rate of carboxylation (*V*_Cmax_) and the maximum electron flow rate supporting RuBP regeneration (*J*_max_). Respiration in the light (*R*_d_) was also calculated as the *y*-axis intercept of the *A*–*C*_i_ response curve. We have to note here that the *V*_Cmax_ and *J*_max_ values reported in our study are adjusted at 25ºC using the temperature functions of Bernacchi *et al.* (2001, 2003). *A*–*C*_i_ curves obtained using dried leaves of each species were used to correct all measurements for CO_2_ leakages (Long and Bernacchi, 2003; Muir *et al.*, 2014).

Incident growth light intensity (*Q*) for each species was measured with an MQ-200 quantum sensor (Apogee Instruments, Inc., Logan, UT, USA) and was then used to calculate the *in situ* electron transport rates from the non-rectangular hyperbola that describes the relationship between photon flux and electron transport (von Caemmerer, 2000) as:

(1) $$J_{situ}=\frac{\;Q_{2}+J_{\max}-\sqrt{{\left(Q_{2}+J_{\max}\right)}^{2}-4\theta Q_{2}J_{\max}}}{2\theta }$$

where *θ* is an empirical curvature factor with an average value of 0·7 (Evans, 1989), *J*_max_ is the maximum electron transport supporting RuBP regeneration and *Q*_2_ is the light utilized by photosystem II (PSII) and is calculated as:

(2) Q2=Q×abs×ΦPSIImax×0·5

(Long and Bernacchi, 2003) where *Q* is the incident photosynthetically active radiation, *abs* is the leaf absorptance and Φ_PSIImax_ is the maximum efficiency of primary photochemistry. A common absorptance value of 0·85 was used in our calculations (von Caemmerer, 2000), and Φ_PSIImax_, which is equivalent to *F*_v_/*F*_m_, was measured with a continuous excitation fluorimeter (see below). Average values of *Q*, *F*_v_/*F*_m_ and *J*_max_ were used for the calculation of *J_situ_* at the species and treatment level.

*In situ* rates of ribulose 1,5-bisphosphate (RuBP) oxygenation (*V*_O_) and carboxylation (*V*_C_) were calculated from the corresponding light curve recordings (i.e. the step of the light curve with an intensity close to that received by each species *in situ*) according to Sharkey (1988) as:

(3) VO=(A+Rd)/[(1/φ) – 0·5]

(4) VC=A+0·5×VO+Rd

(5) $$\varphi =2\times \Gamma^{*}/C_{c}$$

Chloroplastic CO_2_ concentrations (*C*_c_) were calculated from the corresponding *C*_i_ values as:

(6) $$C_{c}=C_{i}–A/g_{m}$$

Mesophyll conductance (*g*_m_) was calculated using the constant *J* modelling method of Harley *et al.* (1992). Five measurements of the RuBP regeneration-limited phase (typically at *C*_i_ values between 50 and 120 Pa) of each *A*–*C*_i_ response curve were used to calculate the photosynthetic linear electron flow rate (*J*) as:

(7) $$J=(A+R_{d})\times \frac{\left[4\times (C_{i}-A/g_{m})+2\times \Gamma^{*}\right]}{(C_{i}-A/g_{m})-\Gamma^{*})\;}$$

Given that *J* is constant when *A* is limited by the regeneration rate of RuBP, the *g*_m_ value that minimizes the variance in *J* was calculated iteratively using the Solver Microsoft Excel add-in (Warren, 2006).

*Γ* ^*^ is the photorespiratory compensation point (i.e. the chloroplastic CO_2_ concentration at which photosynthesis equals photorespiration) and depends on the temperature-sensitive relative affinity of Rubisco for CO_2_ and O_2_. Γ^*^ is considered to be relatively conserved among C_3_ species and is linearly correlated with O_2_ concentration; thus, we assigned standard values of 3·16/2·41 Pa at 20 °C and 21 %/16 % O_2_, respectively, for all five species in our study (Bernacchi *et al.*, 2002).

### Chlorophyll fluorescence measurements

Chlorophyll fluorescence measurements were performed on all three tagged leaves of each plant for a total of 12–18 measurements per species and treatment. After dark-adapting the leaves for 1 h, a Pocket-Pea continuous excitation fluorimeter (Hansatech Instruments Ltd, Kings’ Lynn, Norfolk, UK) was used to capture their fast chlorophyll *a* fluorescence transients. Saturating light (approx. 3500 μmol m^–2^ s^–1^) was provided by a single high intensity red LED (peak at 627 nm), and chlorophyll fluorescence values were recorded from 10 μs to 1 s. Data acquisition rates were 10^5^, 10^4^, 10^3^, 10^2^ and 10 readings per second in the time intervals of 10–300 μs, 0·3–3 ms, 3–30 ms, 30–300 ms and 0·3–1 s, respectively. The cardinal points of the recorded polyphasic fluorescence kinetics [OJIP curves, cardinal points: fluorescence value at 20 μs (*F*_o_), fluorescence value at 300 μs (*F*_300 μs_), fluorescence value at 2 ms (*F*_J_), fluorescence value at 30 ms (*F*_I_) and maximal fluorescence intensity (*F*_m_)] were then used to derive the following parameters according to the JIP-test (Strasser *et al.*, 2004), as extended to include the effect of events related to the final electron acceptors of PSI (Tsimilli-Michael and Strasser, 2008):

(1) *F*_v_/*F*_m_ = (*F*_m_ – *F*_o_)/*F*_m_ or maximum quantum yield of primary photochemistry. *F*_v_/*F*_m_ is a sensitive indicator of stress conditions with typical values of around 0·83 for healthy plants (Bjorkman and Demmig, 1987; Johnson *et al.*, 1993).

(2) *DI*_o_/*RC* = (*ABS/RC*) – (*TR*_o_/*RC*) is the heat dissipation per reaction centre at time zero.

where:

*ABS/RC* = (*M*_o_/*V*_j_)/(1 – *F*_o_/*F*_m_) is the absorption energy flux per PSII reaction centre.

*TR*_o_/*RC* = *M*_o_/*V*_j_ is the trapping per reaction centre at time zero.

and

*M*_o_ = 4 × (*F*_300 ms_ – *F*_o_)/ (*F*_m_ – *F*_o_) is the slope at the origin of the fluorescence rise.

*V*_j_ = (*F*_2 ms_/*F*_o_)/*F*_m_/*F*_o_) is the relative variable fluorescence at 2 ms.

### Statistical analysis

Statistical analysis was performed in R (v·3·1·1). Data were tested for normality and equal variance, and analysed using mixed effects models. Chamber identity was treated as a random effect to identify a possible chamber effect (no chamber effect was detected). Multiple models were run with random and interaction effects. Models were compared using analysis of variance (ANOVA) comparison, and the best fit model was determined using the Akaike information criterion (AIC). One-way ANOVA and Tuckey post-hoc analysis were performed to assess the significance level of the differences between all fixed effects (i.e. species and treatments) from the best fit model.

## RESULTS

**Fig. 1.**
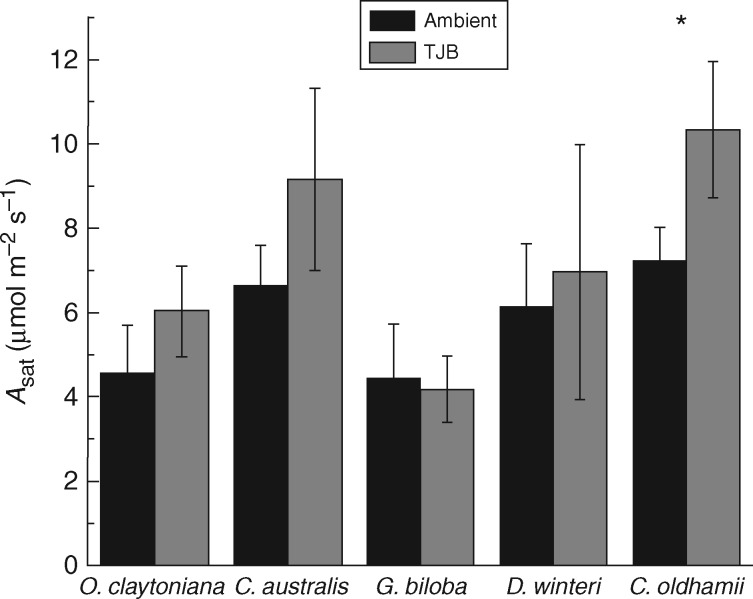
Light-saturated photosynthesis (*A*_sat_) of the test species under ambient (400 ppm CO_2_, 21 % O_2_) and TJB (1900 ppm CO_2_, 16 % O_2_) atmospheric conditions (leaf temperature = 20 ºC). *n* = 3–4 depending on the species. The error bars denote 1 s.d. Asterisks indicate statistically significant differences between treatments for each species (*P* ≤ 0·05).

Light response curves indicated that the *A*_sat_ of the test species did not acclimate uniformly to low [O_2_]:[CO_2_] (Fig. 1). Even though the photosynthetic stimulation was statistically significant only in *C. oldhamii*, the two ferns and the two angiosperms increased their *A*_sat_ by 13·6–43·0 % depending on the species, while the gymnosperm *G. biloba* displayed a small, non-significant decrease (6·1 %). We should note here that *Ginkgo* exhibited a decrease in its light-saturated photosynthetic rate despite the fact that light curves for all species were taken at growth CO_2_ concentration, i.e. nearly 5-fold higher CO_2_ concentration for the TJB treatment plants compared with controls.

This first sign of photosynthetic downregulation under low [O_2_]:[CO_2_] was confirmed by the results of the *A*–*C*_i_ curves (Table 1). Acclimation to low [O_2_]:[CO_2_] led to significant decreases in *V*_Cmax_ and to a lesser extent *J*_max_ values in all test species; however, the decreases were most prominent in *G. biloba*, reaching 64·8 and 57·8 %, respectively, relative to the corresponding values of the control plants (Table 1). As a result of these changes, almost all species exhibited a decreased mean *V*_Cmax_/*J*_max_ ratio under low [O_2_]:[CO_2_] (Table 1), which is indicative of an altered balance between RuBP carboxylation and regeneration typically observed in plants growing at high CO_2_ (Long *et al.*, 2004; Ainsworth and Rogers, 2007; Osada *et al.*, 2010). Yet, we should note that only in three of the species was this decrease statistically significant.

**Table 1.** V_*Cmax*_, J_*max*_, V_*Cmax*_/J_*max*_*ratio,* V_*C*_, V_*O*_, V_*C*_ + V_*O*_, V_*O*_/V_*C*_*ratio and* J_*situ*_*of the species under ambient (400 ppm CO_2_, 21 % O_2_) and TJB (1900 ppm CO_2_, 16 % O_2_) atmospheric conditions*

	Monilophytes				Gymnosperm		Angiosperms			
	*O. claytoniana*		*C. australis*		*G. biloba*		*D. winteri*		*C. oldhamii*	
	Amb	TJB	Amb	TJB	Amb	TJB	Amb	TJB	Amb	TJB
*V*_Cmax_ (μmol m^–2^ s^–1^)	26·7 ± 2·7^a^	13·3 ± 3·0^b^	31·6 ± 1·0^a^	21·1 ± 1·0^b^	47·7 ± 9·3^a^	16·8 ± 3·9^b^	47·9 ± 1·4^a^	20·5 ± 1·0^b^	42·5 ± 5·8^a^	26·9 ± 4·2^b^
*J*_max_ (μmol m^–2^ s^–1^)	61·0 ± 4·6^a^	36·0 ± 9·7^b^	73·7 ± 3·2^a^	54·9 ± 3·0^b^	111·5 ± 21·7^a^	47·0 ± 11·8^b^	93·0 ± 11·1^a^	41·5 ± 2·2^b^	83·6 ± 11·1^a^	61·3 ± 10·8^b^
*V*_Cmax_/*J*_max_	0·44 ± 0·01^a^	0·37 ± 0·01^b^	0·43 ± 0·02^a^	0·38 ± 0·02^a^	0·43 ± 0·02^a^	0·36 ± 0·02^b^	0·52 ± 0·08^a^	0·49 ± 0·01^a^	0·51 ± 0·01^a^	0·44 ± 0·02^b^
*V*_C_ (μmol m^–2^ s^–1^)	4·0 ± 0·4^a^	4·6 ± 0·5^a^	4·8 ± 0·9^a^	5·5 ± 0·3^a^	6·9 ± 0·2^a^	6·0 ± 0·7^a^	5·7 ± 0·5^a^	4·5 ± 1·1^a^	5·1 ± 1·2^a^	5·3 ± 1·4^a^
*V*_O_ (μmol m^–2^ s^–1^)	0·87 ± 0·10^a^	0·15 ± 0·01^b^	1·08 ± 0·15^a^	0·18 ± 0·02^b^	2·75 ± 0·25^a^	0·25 ± 0·12^b^	1·3 ± 0·09^a^	0·13 ± 0·03^b^	1·08 ± 0·28^a^	0·15 ± 0·04^b^
*V*_C_ +*V*_O_ (μmol m^–2^ s^–1^)	4·9 ± 0·5^a^	4·7 ± 0·5^a^	5·9 ± 1·1^a^	5·7 ± 0·4^a^	9·7 ± 0·1^a^	6·2 ± 0·8^b^	7·0 ± 0·6^a^	4·6 ± 1·1^a^	6·2 ± 1·5^a^	5·5 ± 1·5^a^
*V*_O_/*V*_C_	0·22 ± 0·01^a^	0·032 ± 0·002^b^	0·23 ± 0·01^a^	0·033 ± 0·002 ^b^	0·40 ± 0·05^a^	0·041 ± 0·015^b^	0·23 ± 0·01^a^	0·029 ± 0·001^b^	0·21 ± 0·01^a^	0·028 ± 0·001^b^
*J_situ_* (μmol m^–2^ s^–1^)	24·5	18·9	31·9	27·5	62·2	30·9	39·2	27·0	23·2	21·0

*V_Cmax_*, maximum carboxylation rate (25 ºC); *J*_max,_ maximum electron rate supporting RuBP regeneration (25 ºC); *V*_C_, *in situ* carboxylation rate; *V*_O_, *in situ* oxygenation rate; *V*_C_ + *V*_O_, combined oxygenation/carboxylation rate; *J_situ_*, *in situ* electron transport rate. Values are means ± s.d.; *n* = 3–4 depending on species. Different letters for each species denote statistically significant differences between treatments (*P* ≤ 0·05).

**Fig. 2.**
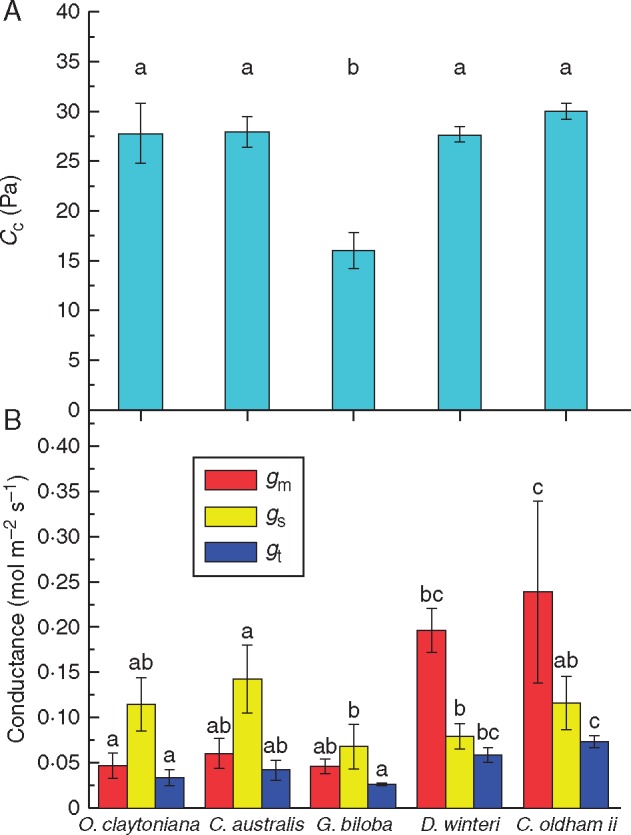
(A) Operational chloroplastic CO_2_ concentrations (*C*_c_) of the test species under ambient atmospheric conditions (400 ppm CO_2_, 21 % O_2_). Data are means ± s.d. from 3–4 measurements. Different letters denote statistically significant differences (*P* ≤ 0·05) between species. (B) Mesophyll conductance (*g*_m_), stomatal conductance at saturating light intensity (*g*_s_) and total conductance [*g*_t_ = (*g*_m_ × *g*_s_)/(*g*_m_ + *g*_s_)] of the test species under ambient atmospheric conditions (400 ppm. CO_2_, 21 % O_2_). *n* = 3–4 depending on species. The error bars denote 1 s.d. Different letters for each parameter denote statistically significant differences (*P* ≤ 0·05) between species.

*In situ* carboxylation rates (*V*_C_) did not display significant changes, despite the decrease in *V*_Cmax_, due to the much higher growth CO_2_ concentration of the TJB treatment (Table 1). As expected, *in situ* oxygenation rates of Rubisco (*V*_O_) diminished due to the very low [O_2_]:[CO_2_] ratio of the growth air mixture in combination with the altered absolute concentrations of both gases (Table 1), yet overall *G. biloba* was the only species to display a significantly decreased combined rate of Rubisco carboxylation/oxygenation under TJB atmospheric conditions (*V*_C_ + *V*_O_, Table 1). Furthermore, *G. biloba* was also the species that displayed the most significant reductions in absolute (*V*_O_) and relative (*V*_O_/*V*_C_) rates of oxygenation primarily as a result of its extraordinarily high rates of Rubisco oxygenation under ambient conditions (Table 1). Indeed, calculation of the total conductance (*g*_t_) from the measured values of *g*_s_ and *g*_m_ revealed that under ambient atmospheric CO_2_*G. biloba* poses substantially higher resistances to CO_2_ diffusion and its photosynthetic machinery operates under significantly lower *C*_c_ compared with the rest of the test species (Fig. 2A, B).

**Fig. 3.**
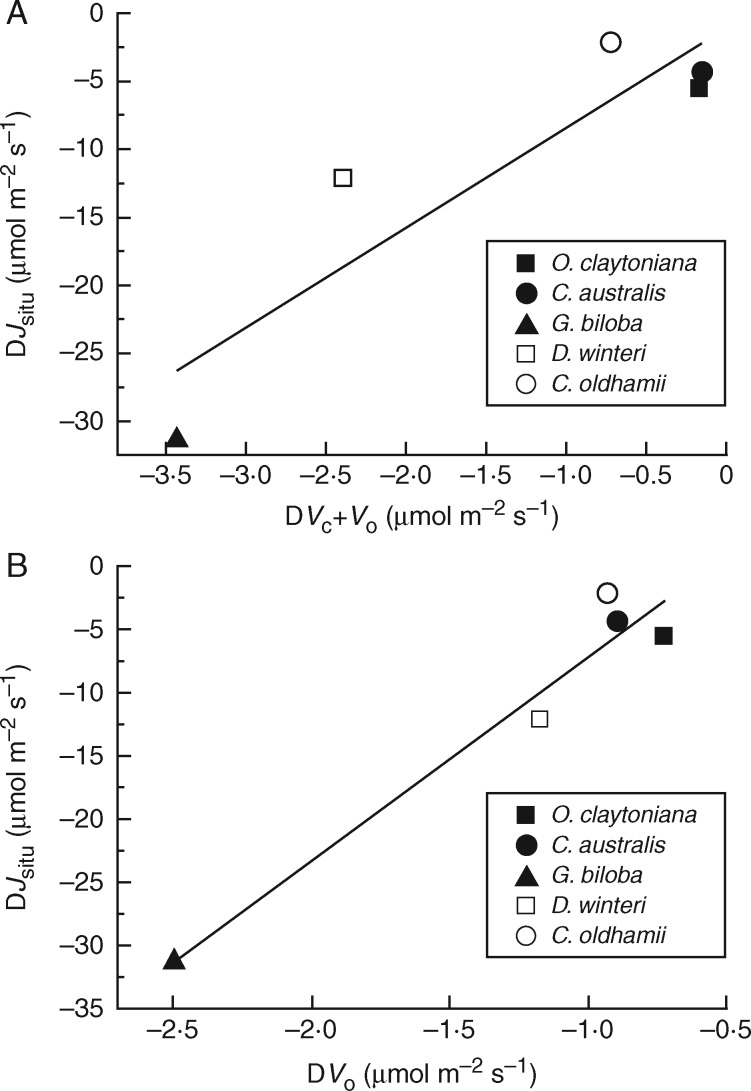
(A) Relationship (*y* = 7·35*x* – 1·03, *r*^2^ = 0·82, *P* = 0·033) between the observed changes in the combined *in situ* oxygenation/carboxylation rate (D*V*_C_ + *V*_O_) and the corresponding changes of the *in situ* electron transport rate supporting RuBP regeneration (D*J_situ_*). Data are differences resulting after subtraction of the mean values of the ambient treatment (400 ppm CO_2_, 21 % O_2_) from the corresponding values of the TJB (1900 ppm CO_2_, 16 % O_2_) treatment, (B) Relationship (*y* = 16·15*x* + 8·98, *r*^2^ = 0·95, *P* = 0·005) between the observed changes in the *in situ* oxygenation rate (D*V*_O_) and the corresponding changes in the *in situ* electron transport rate supporting RuBP regeneration (D*J_situ_*). Data are differences resulting after subtraction of the mean values of the ambient treatment (400 ppm CO_2_, 21 % O_2_) from the corresponding values of the TJB (1900 ppm CO_2_, 16 % O_2_) treatment.

**Fig. 4.**
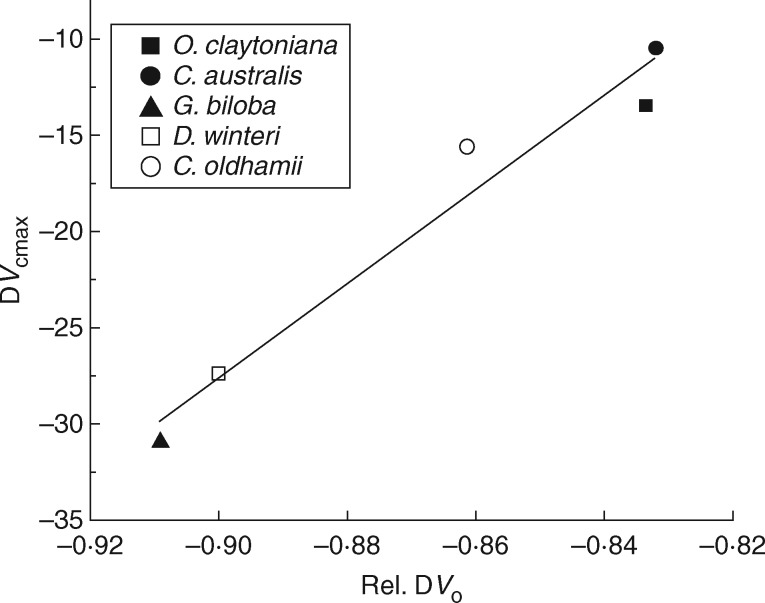
Relationship (*y* = 244*x* + 192, *r*^2^ = 0·95, *P* = 0·003) between the relative observed changes in the *in situ* oxygenation rate [Rel. D*V*_O_  = (*V*_Oamb_ – *V*_OTJB_)/*V*_Oamb_] and the corresponding changes in the maximum rate of carboxylation (D*V*_Cmax_). D*V*_Cmax_ data are differences resulting after subtraction of the mean values of the ambient treatment (400 ppm CO_2_, 21 % O_2_) from the corresponding values of the TJB (1900 ppm CO_2_, 16 % O_2_) treatment.

Ribulose-1,5-bisphosphate is the substrate for both carboxylation and oxygenation reactions of Rubisco, thus the reduction of the combined Rubisco carboxylation/oxygenation rate observed in the TJB treatment *Ginkgo* plants should normally be accompanied by a proportional reduction in the *in situ* rates of electron transport supporting RuBP regeneration (*J_situ_*). Indeed, *J_situ_* values followed a similar pattern, showing small, non-significant changes between control and TJB plants in ferns and *C. oldhamii*, moderate decrease in *D. winteri*, a substantial decrease in *G. biloba* (Table 1) and correlated with the corresponding decreases in *V*_C_ + *V*_O_ (Fig. 3A). It is interesting, however, that when the relatively small changes in *V*_C_ values are ignored and the changes in *J_situ_* are plotted against the corresponding changes in *V*_O_, the correlation becomes more robust (Fig. 3B). In addition, there also seems to be good correlation between the relative decreases in the *in situ V*_O_ [Rel. D*V*_O_  = (*V*_Oamb_ – *V*_OTJB_)/*V*_Oamb_] and the decreases of Rubisco content-dependent *V*_Cmax_ when plants are exposed to the TJB atmospheric treatment (Fig. 4).

**Fig. 5.**
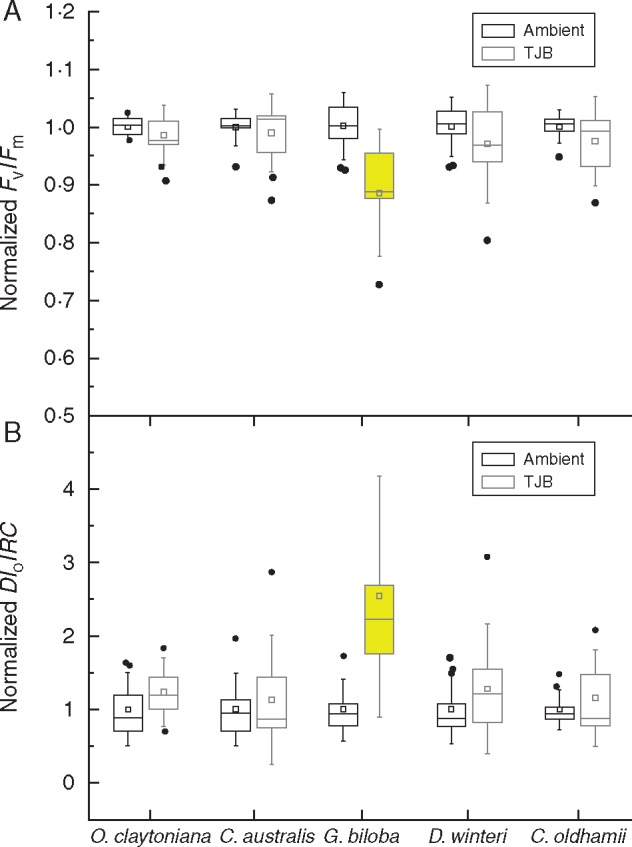
(A) Normalized (relative to ambient values) values of maximum efficiency of primary photochemistry (Normalized *F*_v_/*F*_m_) and (B) normalized values of heat dissipation per reaction centre (Normalized *DI*_o_/*RC*) of the test species under ambient (400 ppm CO_2_, 21 % O_2_, black outline) and TJB (1900 ppm CO_2_, 16 % O_2_, grey outline) atmospheric conditions. In both (A) and (B) the box signifies the distribution of the 25–75 % quartiles, the median and average are represented by a vertical line and an open square within the box, respectively, and the whiskers indicate the s.d. Outliers are represented by filled circles. *n* = 10–18 depending on species. Coloured boxes denote within-species significant differences relative to ambient treatment values (*P* ≤ 0·05).

**Fig. 6.**
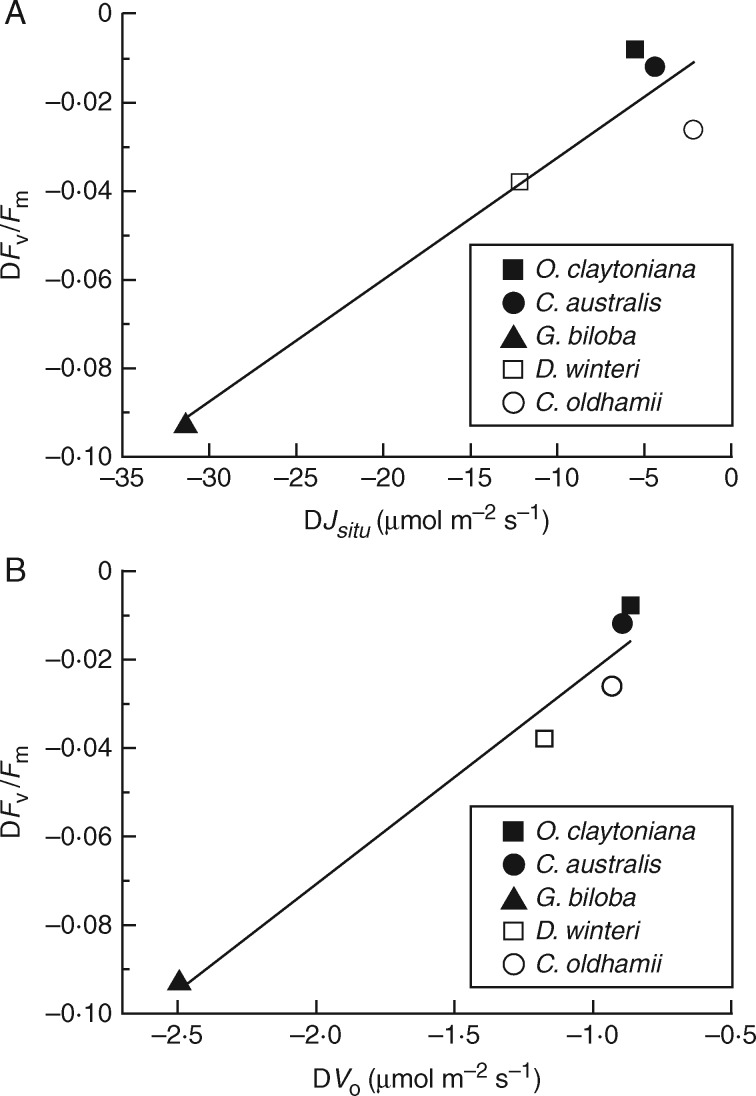
(A) Relationship (*y* = 0·0028*x* – 0·0047, *r*^2^ = 0·91, *P* = 0·011) between the observed changes in the maximum efficiency of primary photochemistry (D*F*_v_/*F*_m_) and the corresponding changes of *in situ* electron transport rate supporting RuBP regeneration (D*J_situ_*). Data are differences resulting after subtraction of the mean values of the ambient treatment (400 ppm. CO_2_, 21 % O_2_) from the corresponding values of the TJB (1900 ppm CO_2_, 16 % O_2_) treatment. (B) Relationship (*y* = 0·048*x* + 0·026, *r*^2^ = 0·96, *P* = 0·004) between the observed changes in the maximum efficiency of primary photochemistry (D*F*_v_/*F*_m_) and the corresponding changes of the *in situ* oxygenation rate (D*V*_O_). Data are differences resulting after subtraction of the mean values of the ambient treatment (400 ppm CO_2_, 21 % O_2_) from the corresponding values of the TJB (1900 ppm CO_2_, 16 % O_2_) treatment.

Exposure to the TJB treatment led to plant group-specific changes in *F*_v_/*F*_m_ (Fig. 5A). Compared with controls, the two fern species maintained high *F*_v_/*F*_m_ values while angiosperms showed moderate, yet non-significant decreases. *Ginkgo biloba* was the only species to display a substantial drop in its *F*_v_/*F*_m_, which indicated that low [O_2_]:[CO_2_] acclimation resulted in partial photoinhibition. Interestingly, changes in *F*_v_/*F*_m_ were found to correlate linearly with changes in both *J_situ_* and *V*_O_ (Fig. 6A, B). Furthermore, in the case of *G. biloba*, the substantial decrease in the maximum quantum yield of primary photochemistry was accompanied by a 2·5-fold increase in the rate of heat dissipation per PSII reaction centre (*DI*_o_/*RC*) (Fig. 5B). This clearly suggested a severely decreased efficiency of photosynthetic light reactions in the low [O_2_]:[CO_2_]-acclimated plants.

## DISCUSSION

Our results clearly demonstrate that *Ginkgo biloba* diverts an extraordinarily high percentage of the ATP and NADPH produced during the photosynthetic light reactions to photorespiratory metabolism when grown under ambient CO_2_ and O_2_ concentrations (Table 1). This is contrary to the pattern observed in the two fern and two angiosperm species studied. Inherent (Yiotis *et al.*, 2010) as well as stress-induced (Flexas *et al.*, 2006) diffusional limitations have been proven to result in a substantial drawdown in *C*_c_ concentrations and high photorespiration rates. Given that the O_2_ concentration in the chloroplast is considered to be about equal to ambient under any circumstance, low stomatal and/or mesophyll conductance to CO_2_ diffusion could lead to decreased chloroplastic CO_2_/O_2_ ratios and thus increased relative rates of *V*_O_. Indeed, our results showed that *G. biloba* poses significantly higher resistance to CO_2_ diffusion compared with the basal angiosperms and ferns of the study (Fig. 2A, B). This seemingly peculiar and extremely low conductance to CO_2_ resembles the decreased conductance observed in plants under stress, but it can be explained by the limited ability of *G. biloba* to adapt to low [CO_2_] by altering its conductance to CO_2_ diffusion. This is supported by recent evidence that the dynamic range of stomatal conductance of *Ginkgo* is constrained due to its low anatomical *G*_max_ (McElwain *et al.*, 2016) and the fact that the mesophyll conductance of gymnosperms is generally low (Flexas *et al.*, 2012).

Yet, *C*_c_ does not depend solely on a species’ resistance to CO_2_ diffusion. *C*_c_ is a function of both *g*_t_ and *A* (von Caemmerer, 2000); thus, in spite of the anatomical and/or biochemical restrictions limiting the *g*_s_ and *g*_m_ of *G. biloba*, the species could still increase its operational *C*_c_ by photosynthesizing at lower rates. Since the light-saturated photosynthesis of *G. biloba* is clearly limited by its Rubisco carboxylation capacity, a reduction in the enzyme’s content could facilitate an increase in the operational *C*_c_ values. It is apparent that such a reallocation of nitrogen from Rubisco to other proteins would decrease the relative photorespiratory carbon losses under low [CO_2_]. Although photorespiration was initially considered a wasteful evolutionary relic resulting from a lack of selective pressure when Rubisco was selected by photosynthetic bacteria as their primary carboxylase, recent evidence has demonstrated the importance of photorespiration in photoprotection (Osmond and Grace, 1995; Kozaki and Takeba, 1996; Niyogi, 2000; Takahashi *et al.*, 2007) and nitrogen assimilation (Somerville and Ogren, 1980; Rachmilevitch *et al.*, 2004; Heldt, 2005; Bloom *et al.*, 2010; Bloom, 2015). Interestingly, our results show that there is good correlation between the decreases in *V*_O_ and the Rubisco content-dependent *V*_Cmax_ (Fig. 4). Moreover, due to the sheer amount of leaf nitrogen invested in Rubisco (Evans, 1989), we could argue that *V*_Cmax_ generally reflects plant nitrogen content and nitrogen assimilation capacity (Walker *et al.*, 2014). Concomitantly, the correlation between the relative decreases in *V*_O_ and *V*_Cmax_ seems to support the proposed link between photorespiration and nitrogen assimilation.

**Fig. 7.**
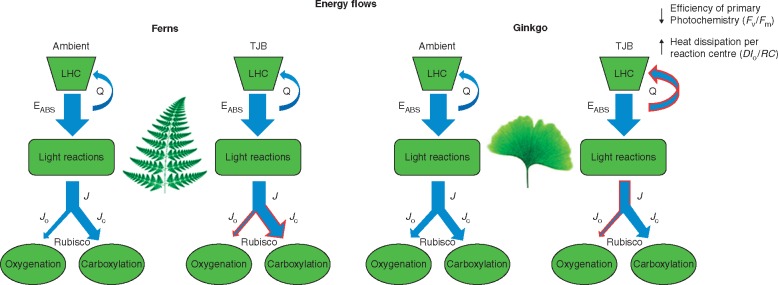
Schematic model depicting the changes in the energy flows of *Ginkgo* and ferns when acclimated to TJB atmospheric conditions. The thickness of the arrows is representative of the relative magnitude, and the flows that change under low [O_2_]:[CO_2_] are outlined with red colour. LHC, light-harvesting complex, E_ABS_, absorbed energy; Q, heat dissipation; *J*, photosynthetic electron flow; *J*_O_, photosynthetic electron flow supporting photorespiratory metabolism; *J*_C_, photosynthetic electron supporting photosynthesis; *DI*_o_/*RC*, heat dissipation per reaction centre, *F*_v_/*F*_m_, maximum efficiency of primary photochemistry.

Plants have a complex control mechanism that adjusts the rate of the ‘light photosynthetic reactions’ and the combined rate of photosynthesis and photorespiration so that they match each other (Sharkey, 1990; Zhang and Portis, 1999; Andersson, 2008; Parry *et al.*, 2008); however, stress conditions can slow down the rate of the Calvin-Benson-Bassham cycle, thus generating a potentially harmful imbalance. In these cases, photorespiration is believed to act as a safety valve, quenching the excess absorbed light energy by consuming the produced ATP and NADPH that cannot be used for carbon assimilation. Exposure to a TJB treatment, however, diminished photorespiration (Table 1; Fig. 7) and in conjunction with the stable or decreased *in situ V*_C_ (Table 1; Fig. 7) led to a drop in the demand for RuBP regeneration, which is reflected in the corresponding drops in operational *J_situ_* (Table 1; Figs. 3A, B and 7).

Under these conditions of reduced capacity for photosynthetic and photorespiratory quenching of absorbed light energy, the electron transport chain of the light reactions becomes over-reduced and plants are forced to increase the efficiency of energy quenching through heat dissipation in order to avoid permanent photodamage (Demmig-Adams and Adams, 1996; Horton *et al.*, 1996; Müller *et al.*, 2001; Lambrev *et al.*, 2012). The amplitude of this increase depends on the amount of excess absorbed energy or, in our case, the drop in the capacity for photosynthetic and photorespiratory quenching, assuming no significant changes in leaf absorption. Indeed, under low [O_2_]:[CO_2_], *G. biloba* displayed the highest increase in the heat dissipation per reaction centre (*DI*_o_/*RC*) compared with the rest of the species, with ferns and angiosperms only showing negligible changes (Figs 5B and 7). Even so, it appears that the increased efficiency of heat dissipation is not adequate to alleviate photoinhibition, as revealed by the significant decrease of *F*_v_/*F*_m_ in *G. biloba* under TJB atmospheric conditions (Figs 5A, 6A, B and 7).

Our results do not allow us to identify the increased rate of photorespiration in *G. biloba* either as proof of the species’ reduced fitness to current low atmospheric CO_2_ or as an evolutionary strategy aiming to enhance the species’ persistence to stress and/or nitrogen assimilation capacity. Yet, it is apparent that exposure to a low [O_2_]:[CO_2_] atmosphere and subsequent diminishment of the photorespiratory sink for photosynthetic electron flow severely affects the species’ competitiveness, especially when compared with the fern species, which display a high level of photosynthetic plasticity. In contrast to *G. biloba*, ferns and angiosperms displayed a remarkable adaptability of their physiology to TJB atmospheric conditions. Ferns’ adaptive decreases in *V*_Cmax_ and *J*_max_ (Table 1) were counterbalanced by the increased CO_2_ concentration in such a way that both the *in situ V*_C_ and *A*_sat_ showed an increasing, yet non-statistically significant, trend (Table 1; Fig. 1). In addition, the absence of changes in the heat dissipation per reaction centre (*DI*_o_/*RC*) and the maximum efficiency of primary photochemistry (*F*_v_/*F*_m_) under nearly non-photorespiratory conditions further highlights their ability to acclimate their physiology effectively to low [O_2_]:[CO_2_] (Figs 5A, B and 7).

We acknowledge that an increase in global temperature and high atmospheric CO_2_-induced decreased transpiration of the broadleaved Ginkgoales must have played a role in their decline across the TJB (McElwain *et al.*, 1999). However, the present work provides an additional mechanism that may have contributed to the near extinction of Ginkgoales and to the proliferation of ferns evident in the TJB fossil record (Fowell and Olsen, 1993; Olsen *et al.*, 2002; McElwain *et al.*, 2007). Our study focused exclusively on innate differences in the photosynthetic plasticity and did not investigate the anatomical adaptations of leaves grown under a TJB atmosphere. Yet, we would like to note that after >100 million years of plant evolution and despite the plummeting of atmospheric CO_2_ levels, gymnosperms still display relatively low values of stomatal and mesophyll conductance, even though higher values would have enabled them to maintain higher photosynthetic rates and/or higher photosynthetic nitrogen use efficiency (Flexas *et al.*, 2012; McElwain *et al.*, 2016). Based on this apparent reduced anatomical/physiological adaptability of gymnosperms, we believe that the responses of present-day *G. biloba* to a TJB treatment are likely to resemble those of Ginkgoales present during the TJB. Overall, our study stresses the importance of differences in the physiological plasticity of different plant groups in shaping evolutionary patterns under fluctuating atmospheric O_2_ and CO_2_. Thus, in light of ongoing rapid increases in atmospheric CO_2_ levels, we argue that further investigation of the innate physiological characteristics and constraints of different plant groups are of considerable significance as we are likely to be on the verge of major shifts in the composition of plant communities.

## SUPPLEMENTARY DATA

Supplementary data are available online at https://academic.oup.com/aob and consist of the following. Table S1: classification, size of pots, compost mix and fertilizer used for each of the studies. Table S2: mean values ± s.d. of growth conditions in the chambers used in the study.

## Supplementary Material

Supplementary Data
