# Supplementary material for: Differences in the photosynthetic plasticity of ferns and Ginkgo grown in experimentally controlled low [O2]:[CO2] atmospheres may explain their contrasting ecological fate across the Triassic–Jurassic mass extinction boundary
Source: Ann Bot. 2017 Mar 11;119(8):1385–95. doi: 10.1093/aob/mcx018 (PMC5604595; doi:10.1093/aob/mcx018)
Supplement: Supplementary Data [file mcx018_Supp.zip › mcx018-suppl_data/aob-16731-s02.rtf]

Supporting Information 

Table S2 Mean values ± standard deviation of growth conditions in the chambers used in the study. Note that CO2 values are averages of day and night CO2 (respiration caused increases above set point at night) and intervals of entering the chambers for watering and gas exchange measurements.
Treatment
	Chamber ¹	CO2 (ppm)	O2 (%)	Light           (ìmol m-2 s-1)
(µmol m-2 s-1)	Humidity (%) 

 (%)	Temperature (°C)
	
						Day (°C)	Night (°C)	
Control	7	426 ± 73	21.3 ± 0.2	583 ± 35	63.5 ± 2.9	20.0 ± 0.1	15.0 ± 0.1	
Control	8	446± 69	21.2 ± 0.3	598 ± 15	64.2 ± 2.0	20.0 ± 0.1	15.0 ± 0.1	
TJB	4	1894± 72	16.5 ± 1.0	590 ± 34	63.0 ± 2.6	20.0 ± 0.2	15.0 ± 0.3	
TJB	5	1897± 41	16.4 ± 0.9	594 ± 17	64.7 ± 1.5	20.0 ± 0.3	15.0 ± 0.4	
