# Supplementary material for: Differences in the photosynthetic plasticity of ferns and Ginkgo grown in experimentally controlled low [O2]:[CO2] atmospheres may explain their contrasting ecological fate across the Triassic–Jurassic mass extinction boundary
Source: Ann Bot. 2017 Mar 11;119(8):1385–95. doi: 10.1093/aob/mcx018 (PMC5604595; doi:10.1093/aob/mcx018)
Supplement: Supplementary Data [file mcx018_Supp.zip › mcx018-suppl_data/aob-16731-s01.rtf]

Supporting Information 

Table S1 Classification, size of pots, compost mix and fertilizer used for each of the study species. 
		Monilophytes		Gymnosperm		Angiosperms	
Order		Cyatheales	Osmundales		Ginkgoales		Canellales	Chloranthales	
Family		Cyatheaceae	Osmundaceae		Ginkgoaceae		Winteraceae	Chloranthaceae	
Genus		Cyathea	Osmunda		Ginkgo		Drimys	Chloranthus	
Species		C. australis	O. claytoniana		G. biloba		D. winteri	C. oldhamii	
Pot size (l)		3	4		4		4	4	
Compost mix (v:v)		90:10
Shamrock multi-purpose compost:vermiculite	60:40
Rotted bark:Shamrock multi-purpose compost		58:25:17
Loam:Peat:Sand		58:25:17
Loam:Peat:Sand	80:20
Shamrock multi -purpose compost:vermiculite	
Fertilizer		2 Osmocote plugs (5-6 months)	10g/l Osmocote (12-14 months)		10g/l Osmocote (12-14 months)		10g/l Osmocote (12-14 months)	10g/l Osmocote (12-14 months)	
Osmocote (Scotts, Marysville, OH, USA) composition is 15 % N, 10 % P2O5, 10 % K2O, 2 % MgO, plus trace elements
